# Dietary Vitamin A Intake and Circulating Vitamin A Concentrations and the Risk of Three Common Cancers in Women: A Meta-Analysis

**DOI:** 10.1155/2022/7686405

**Published:** 2022-11-02

**Authors:** Xiaoyong Han, Rangyin Zhao, Yongfeng Wang, Haizhong Ma, Miao Yu, Xiaohong Chen, Dongzhi Zhang, Shixun Ma, Bin Liu, Hui Cai

**Affiliations:** ^1^Graduate School, Ningxia Medical University, Yinchuan, Ningxia 750004, China; ^2^Gansu Provincial Hospital, Lanzhou, Gansu 730000, China; ^3^Key Laboratory of Molecular Diagnostics and Precision Medicine for Surgical Oncology in Gansu Province, Gansu Provincial Hospital, Gansu 730000, China; ^4^Gansu University of Chinese Medicine, First Clinical Medical College, Lanzhou, Gansu 730000, China; ^5^First Clinical College of Medicine, Lanzhou University, 1st West Donggang R.D., Lanzhou 730000, China; ^6^Key Laboratory of Evidence-Based Medicine and Knowledge Translation of Gansu Province, Lanzhou, Gansu 730000, China; ^7^NHC Key Laboratory of Diagnosis and Therapy of Gastrointestinal Tumor, Gansu Provincial Hospital, Lanzhou 730000, China

## Abstract

**Background:**

According to relevant clinical research, dietary and circulating antioxidants vitamin A are connected with the risk of breast, cervical, and ovarian cancer in women. However, there was inconsistency between the findings. We completed this meta-analysis at the right moment to address this contradiction of the problem.

**Methods:**

Web of Science, Embase, and PubMed databases were searched using the proposed search strategy and filtered using the inclusion and exclusion criteria as well as the NOS quality score. As of May 2022, low intake or low concentration was used as a control, and odds ratio (OR) or relative risk (RR) and ninety-five percent confidence intervals (95% CI) were extracted for high intake. Stata 12.0 was used to process the data.

**Results:**

Our meta-analysis included a total of 49 studies, 29 on breast cancer, 10 on ovarian cancer, and 10 on cervical cancer. There were 38 case-control studies included, with 25,363 cases and 42,281 controls; there were 11 cohort studies included, 1,334,176 individuals were followed up, and finally 9496 obtained cancer. The pooled OR value results were as follows: diet or supplements (OR = 0.83, 95% CI 0.76-0.90, *I*^2^ = 56.1%) and serum or plasma (OR = 0.96, 95% CI 0.86-1.09, *I*^2^ = 29.5%). Subgroup analyses were performed according to cancer type, diet or supplements, serum or plasma, study type, and geographic regions.

**Conclusions:**

In North American and Asian populations, high dietary consumption of vitamin A or supplements decreases the incidence of three cancers in women, with breast and ovarian cancers being more significant. However, high circulating vitamin A concentrations were not significantly connected with the risk of the three malignancies.

## 1. Introduction

With the development of science and technology and the progress of society, women's health has been paid more and more attention by people. However, the incidence and mortality of malignant tumors have gradually increased and become a major killer endangering human health [1, 2]. Among them, ovarian, breast, and cervical cancer are important cancers that endanger women and bring great pressure to health institutions and medical resources [2–5]. Since this century, medical technology has advanced significantly. Comprehensive treatments have enhanced the efficacy of malignant tumors, but the prognosis is still poor, and early diagnosis is still difficult [6–8]. The etiology of cancer is mainly affected by the environment and living habits. Diet, age, obesity, lack of physical exercise, adverse emotions, HPV infection, and family history are all risk factors for the above tumors [9–11]. Therefore, how to prevent these tumors by changing dietary habits and lifestyle is our research focus.

According to studies, excessive generation of reactive oxygen species (ROS) in the body has been linked to the emergence and progression of a number of chronic and degenerative disorders, including Alzheimer's disease, cardiovascular disease, digestive system diseases, and malignant tumors [12, 13]. Normally, in healthy individuals, antioxidants modulate the concentration of ROS to keep it fluctuating within a certain range [14, 15]. The chance of developing malignant tumors and other disorders rises when antioxidant deficiency results in increased ROS concentration, which increases the body's susceptibility to oxidative stress [15]. Therefore, antioxidants are critical in counteracting oxidative stress and regulating ROS concentration [16]. Antioxidants can be taken through endogenous supplementation or dietary intake. It has been found that some vitamins, including vitamin A and vitamin D, can reduce oxidative stress damage, prevent disease occurrence, and control disease progression [17–19]. Among them, vitamin A is a crucial member of the vitamin family. Vitamin A is a fat-soluble vitamin that involves retinol and retinol derivatives (retinoic acid, retinyl esters, and retinaldehyde) [20, 21]. Vitamin A deficiency is common in the population because it cannot be synthesized by itself in the body and must be actively obtained from food [22, 23]. Vitamin A has antioxidant and gene transcriptional regulation and plays a key role in cell differentiation, growth, proliferation, and immunity [24–26]. Vitamin A insufficiency can cause diseases such as decreased immunity, night blindness, dry skin, and diarrhea [19, 27]. According to related studies, vitamin A insufficiency raises the incidence of certain cancers and deserves our further exploration [23, 28, 29].

Since the new century, with the deepening of research on the role and mechanism of antioxidants, the great medical prospects of antioxidants have been revealed and attention has been paid to the association between antioxidant intake and various cancers [30]. Vitamin A deficiency is correlated to an increased incidence of certain malignancies, such as breast cancer [31], lung cancer [32], skin cancer [33], cervical cancer [34], gastric cancer [35], liver cancer [29], and ovarian cancer [36]. Today, we will look at the link between vitamin A consumption and circulating concentrations and breast, cervical, and ovarian cancer. Some studies suggest that its intake and circulating concentration are negatively correlated with the risk of these tumors [34, 37, 38], and some studies show no significant correlation [39–41]. Thus, we completed the meta-analysis in order to synthesize related research and draw more reliable conclusions to guide cancer prevention and promote women's health.

## 2. Methods and Materials

### 2.1. Search Strategy for Literature

Utilizing the Embase, Web of Science, and PubMed English databases, two writers (Han X and Zhao R) independently searched the literature for vitamin A and human breast, cervical, and ovarian cancer risk. The key search terms were as follows: “Vitamin A” or “Retinol” or “All-Trans-Retinol” or “All Trans Retinol” or “Nutrients” paired with “Breast cancer” or “Cervical cancer” or “Ovarian cancer”. The search period was from inception to May 2022. In addition, to avoid possibly overlooked papers, we carefully examined references from pertinent reviews, meta-analyses, and so on. The language included in this study was restricted to English. The third writer resolved the search debate between the two writers.

### 2.2. Criterion for Inclusion and Exclusion

The following standards were applied to determine inclusion studies: (1) the patients were definitely diagnosed with breast cancer or ovarian or cervical cancer; (2) the types of included studies were prospective cohort studies and case-control studies; (3) the subjects of the studies were human clinical studies, excluding cell and animal trials; (4) the correlations between circulation or dietary vitamin A levels and supplement doses and the incidence of breast, ovarian, and cervical cancer were the connections of interest; and (5) containing data indicating the risk of the disease: odds ratio (OR) or relative risk (RR) and ninety-five percent confidence intervals (95% CI). We applied the following exclusion criteria: (1) reviews or meetings or letters to the editors or abstracts, (2) studies on animals, (3) repeat studies, (4) research into other malignancies, (5) lack of data on RR or OR, and (6) other kinds of vitamins.

### 2.3. Data Extraction and Quality Assessment

Two writers completed quality assessment and extracted pertinent data on the included research, respectively. The following basic information was included: the first author's name, publishing year, region, age, cancer type, kind of study design, sample size, OR or RR, and 95% CI, covariates involved in correction. The differences between the two writers Han X and Zhao R were discussed and decided by other writers. The NOS score was utilized to judge the quality of each article, and studies with scores more than or equal to 6 points were eligible for inclusion in this meta-analysis.

### 2.4. Analysis of Data

From the included studies, the cancer risk HR, OR, or RR of the highest intake was extracted one by one against the lowest intake (or the lowest concentration in the circulation). In the original study, prospective cohort studies mostly used RR values or HR values to assess the risk of morbidity and case-control studies mostly used OR values to evaluate the risk of morbidity. Since the differences among the three are small, all risk results are presented as ORs to better combine and calculate the study results. In addition, we performed *Q* and *I*^2^ tests to analyze the heterogeneity of the studies. If the *Q*-test (PQ), *P* value < 0.1, or *I*^2^ > 50%, indicating significant heterogeneity among the research, a random-effects model was utilized. On the contrary, a fixed-effects model was utilized. Forest plots were used to show the combined outcomes of this meta-analysis, and Begg's funnel plot and Begg's test were used to evaluate the publication bias of the studies. Finally, the stability of the combined data was tested using sensitivity analysis. All the above calculations were analyzed using the Stata 12.0 software for Windows, and *P* < 0.05 was regarded as statistically significant.

## 3. Results

### 3.1. Screening Procedures for Studies Meeting Criteria

**Figure 1:**
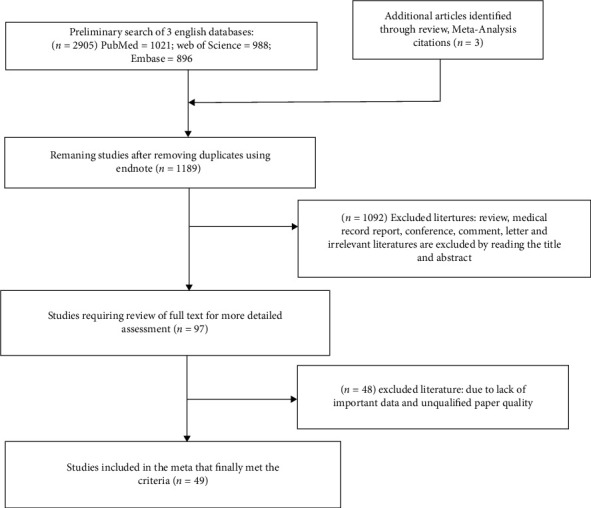
Flow diagram of this meta-analysis.

Perform related literature search in 3 major databases using the specified search methods: total (*n* = 2905), including PubMed (*n* = 1021), Web of Science (*n* = 988), and Embase (*n* = 896). Endnote was used to remove duplicates leaving 1189 articles. After reviewing the article titles and abstracts, 1092 were excluded because they were not related to the study subjects, article type, or study purpose. 97 full texts needed to be downloaded, of which 48 were excluded after initial examination because of unqualified article quality or lack of important data. Finally, 49 publications that fully satisfied the inclusion criteria and were of sufficient quality were included in our meta-analysis.  Figure 1 depicts the retrieval procedure mentioned previously.

### 3.2. Characteristics of the Included Studies

The key features of the 49 studies included in the meta-analysis are shown in Table 1. With regard to breast cancer, 29 studies were included, including 9 cohort studies; 937,252 people were followed up for 5-15 years, and finally 8,146 people had breast cancer; 20 case-control studies, involving 18,915 cases and 27,660 controls; 19 studies on diets or supplements, 6 studies on serum, and 7 studies on plasma. For cervical cancer, 10 studies were included, including 1 cohort study; 299,649 people were followed up for 5-15 years, and finally 1,070 people had cervical cancer; 9 case-control studies involving 2682 cases and 5,305 controls; 9 studies on diets or supplements, 2 studies on serum, and 1 study on plasma. For ovarian cancer, 10 studies were included, including 1 cohort study with 97,275 individuals followed up for 8-15 years resulting in 280 ovarian cancers; 9 case-control studies with 3,766 cases and 9,316 controls; 10 studies on meals or supplements and 1 study on plasma. Studies were published between 1988 and 2021. All included research was adjusted for multivariate covariates, such as age, smoking, sex, drinking, relevant genetic history of cancer, marriage and childbearing history, menstrual history, and energy intake. The NOS was graded on a scale of 6 to 8.

### 3.3. Association of Diet Consumption of Vitamin A or Supplements with Risk of Three Cancers in Women

**Figure 2:**
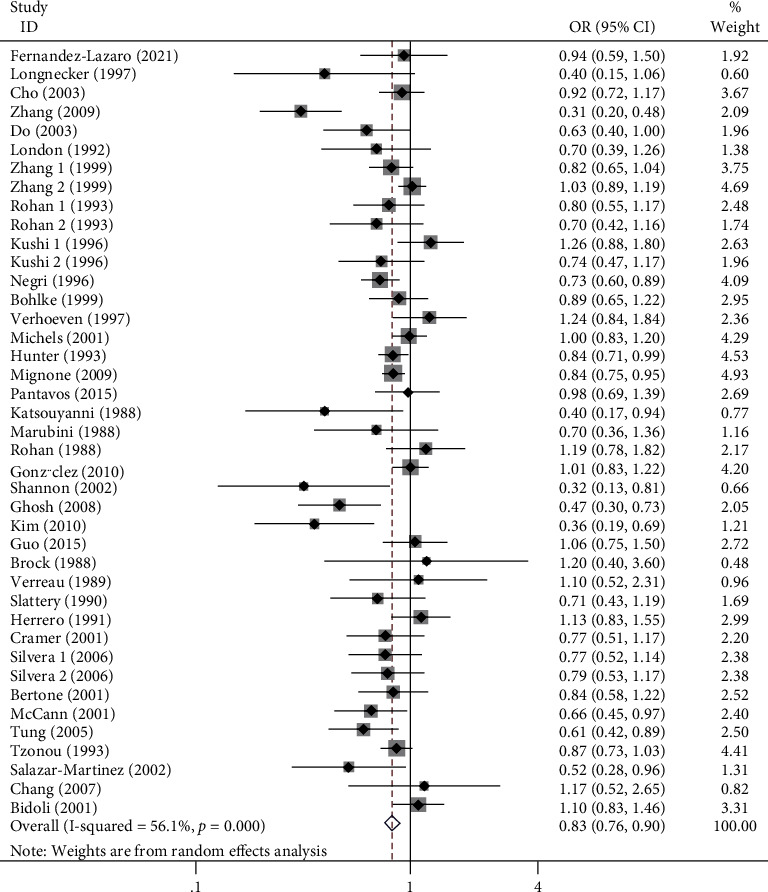
Forest plot of high dietary intake of vitamin A or supplements and risk of breast, cervical, and ovarian cancer in women.

**Figure 3:**
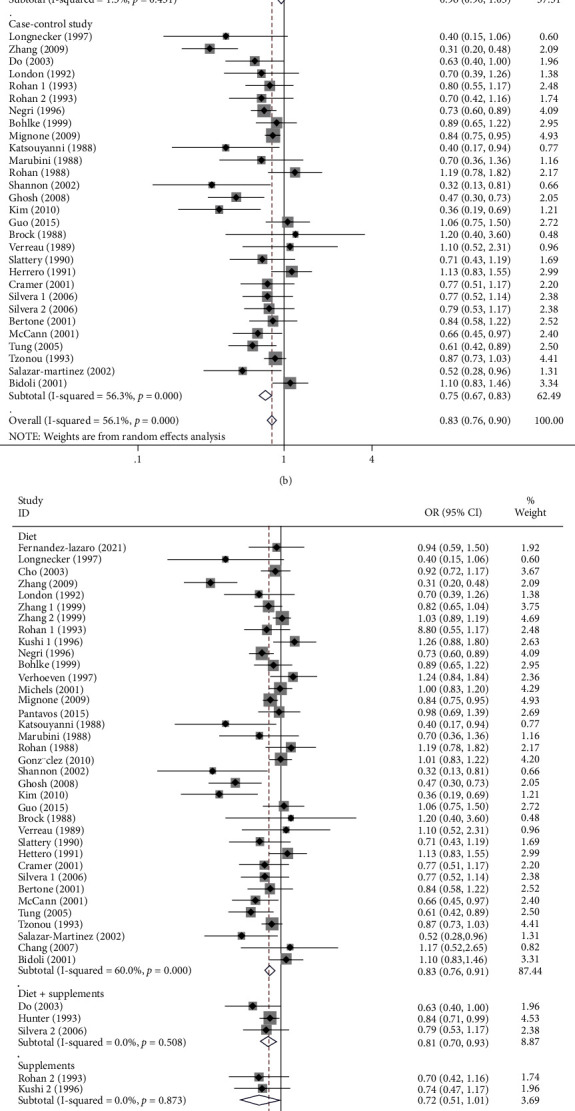
Subgroup analysis of high dietary vitamin A or supplement intake and risk of breast, cervical, and ovarian cancer in women. (a) Subgroup analysis of tumor types; (b) subgroup analysis of study types; (c) subgroup analysis of dietary and supplements; (d) subgroup analysis of geographical location.

In terms of diet or supplements, a sum of 41 sets of data from 37 researches was considered. Forest plot results showed that high intake of dietary vitamin A or supplements significantly (*p*_*t*_ = 0.001) reduced a woman's risk of three cancers by 17% (OR = 0.83, 95% CI 0.76-0.90; Figure 2). Because of the high heterogeneity (*I*^2^ = 56.1%, *P* < 0.001), we chose a random-effects model for pooling. We subgroup analyzed the kind of cancer and discovered a significant inverse relationship between high vitamin A consumption and breast cancer incidence (OR = 0.84, 95% CI 0.76-0.93; Figure 3(a)) and ovarian cancers (OR = 0.81, 95% CI 0.72-0.92; Figure 3(a)) and a nonsignificant inverse association with the cervical cancer risk (OR = 0.77, 95% CI 0.59-1.02; Figure 3(a)). In addition, we subgroup analyzed the type of study; cohort studies (OR = 0.96, 95% CI 0.90-1.03; Figure 3(b)) found that a higher vitamin A consumption might lower the incidence of three malignancies marginally but not considerably, and case-control studies (OR = 0.75, 95% CI 0.67-0.83; Figure 3(b)) found that higher consumption can significantly lower the incidence of three cancers. Then, we subgroup analyzed according to diet or supplements, and high dietary vitamin A (OR = 0.83, 95% CI 0.76-0.91; Figure 3(c)) consumption and diet plus supplements (OR = 0.81, 95% CI 0.70-0.93; Figure 3(c)) were all able to significantly reduce the incidence of three cancers in women, and supplements (OR = 0.72, 95% CI 0.51-1.01; Figure 3(c)) lower their risk but not significantly. Finally, we made subgroup analysis based on the geographic region of the study area, including Asia (OR = 0.50, 95% CI 0.29-0.86; Figure 3(d)), Europe (OR = 0.91, 95% CI 0.82-1.01; Figure 3(d)), North America (OR = 0.83, 95% CI 0.75-0.91; Figure 3(d)), and Oceania (OR = 1.19, 95% CI 0.80-1.77; Figure 3(d)). The findings revealed that a higher diet and supplement consumption considerably lowered the incidence of all three malignancies in women in North America and Asia, but no significant association was found in Oceania and Europe.

### 3.4. The Relationship between Circulating Vitamin A Concentrations and the Risk of Three Malignancies in Women

**Figure 4:**
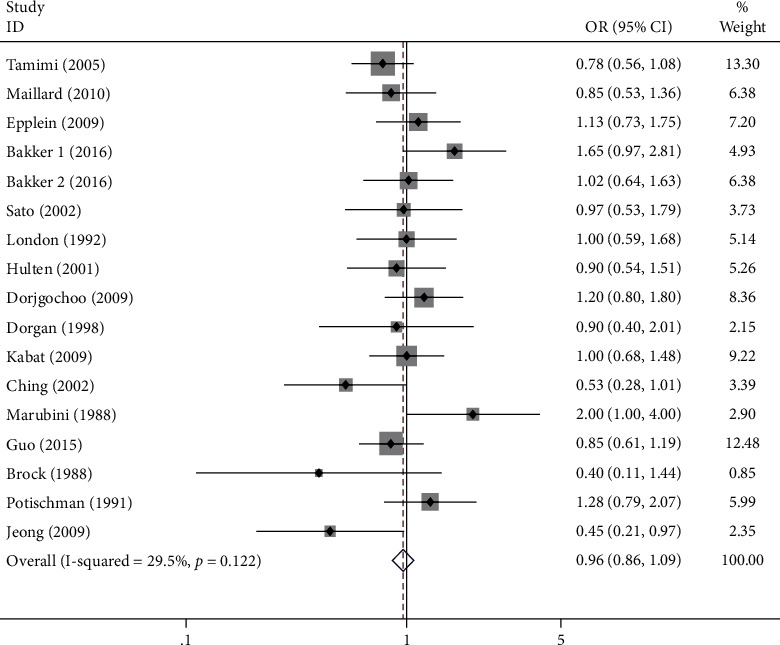
Forest plot of circulating high levels of vitamin A and risk of breast, cervical, and ovarian cancer in women.

**Figure 5:**
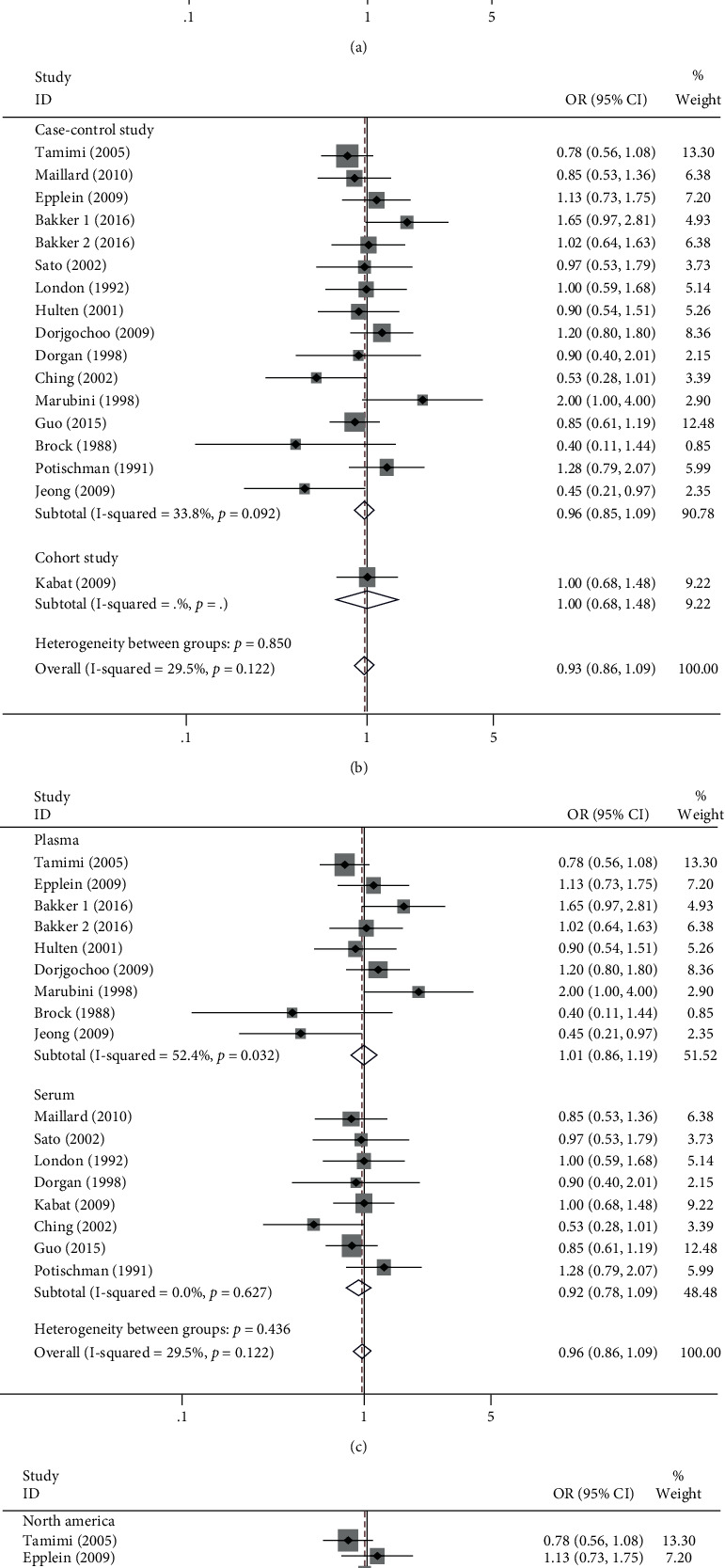
Subgroup analysis of circulating high levels of vitamin A and risk of breast, cervical, and ovarian cancer. (a) Subgroup analysis of tumor types; (b) subgroup analysis of study types; (c) subgroup analysis of serum and plasma; (d) subgroup analysis of geographical location.

Regarding circulation, we included a total of 16 studies with 17 sets of data. Forest plot results showed that high concentrations of circulating vitamin A (OR = 0.96, 95% CI 0.86-1.09, *p*_*t*_ = 0.552; Figure 4) were not associated with cancer risk in women. Since there was no significant heterogeneity (*I*^2^ = 29.5%, *P* = 0.122), we chose the fixed-effects model to combine effect sizes. Our subgroup analysis by types of malignant tumor found that high concentrations of circulating vitamin A were not significantly associated with the incidence of breast (OR = 1.00, 95% CI 0.87-1.14; Figure 5(a)) and cervical malignant tumor (OR = 0.93, 95% CI 0.71-1.12; Figure 5(a)). Furthermore, we subgroup analyzed by type of study, and the case-control studies (OR = 0.96, 95% CI 0.85-1.09; Figure 5(b)) found that high concentrations of circulating vitamin A were not significantly associated with cancer incidence in women. Then, we subgroup analyzed serum or plasma concentrations and found no significant association between high serum vitamin A concentrations (OR = 0.92, 95% CI 0.78-1.19; Figure 5(c)) and high plasma vitamin A concentrations (OR = 1.01, 95% CI 0.86-1.19; Figure 5(c)) and the risk of malignant tumor in women. Finally, we performed a subgroup analysis according to geographic location at the study site, and circulating vitamin A concentrations were not associated with the risk of these three cancers in North American (OR = 0.97, 95% CI 0.82-1.16; Figure 5(d)), European (OR = 1.12, 95% CI 0.89-1.42; Figure 5(d)), and Asian women (OR = 0.90, 95% CI 0.71-1.15; Figure 5(d)) but were significantly negatively associated with the incidence of these three cancers in Oceania women (OR = 0.50, 95% CI 0.28-0.89; Figure 5(d)).  Table 2 summarizes the above meta-analysis findings.

### 3.5. Publication Bias and Sensitivity Analysis

**Figure 6:**
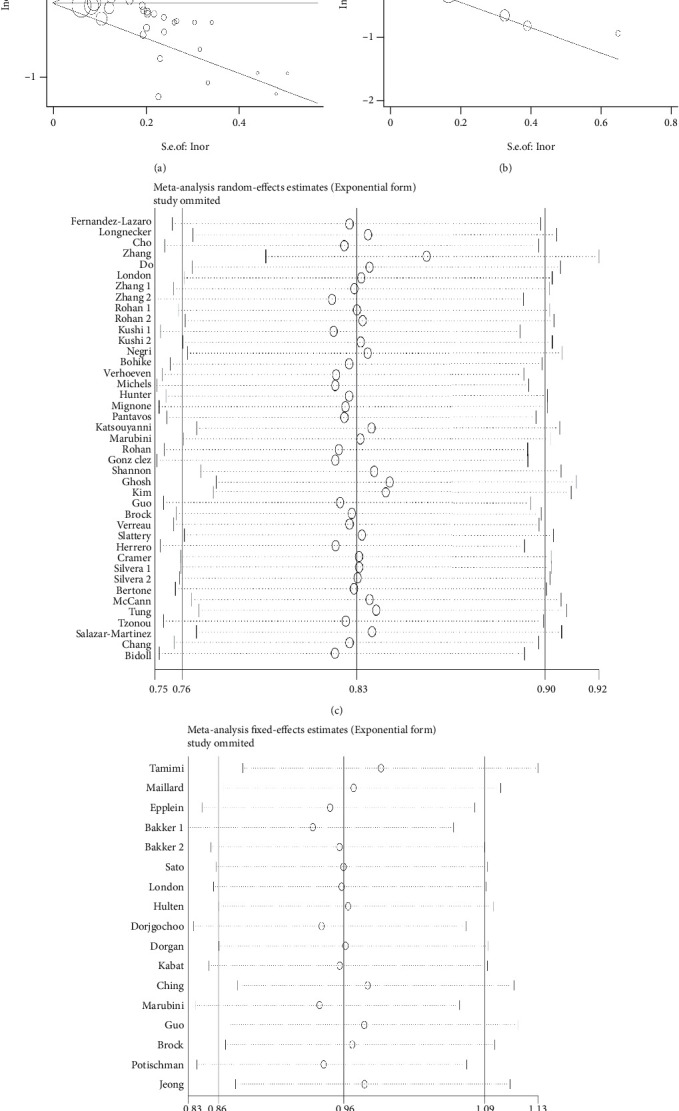
Begg's funnel plot and sensitivity analysis for publication bias test. (a) Begg's plot of combined results of diet or supplements; (b) Begg's funnel plot of pooled circulating results; (c) sensitivity analysis of combined results of diet or supplements; (d) sensitivity analysis of pooled circulating results.

In order to determine if there was publication bias in the combined data, we utilized Begg's funnel plot and Begg's test. Firstly, the combined results of diet and supplement and risk of cancer were evaluated. Begg's funnel plot seemed to have slight asymmetry (Figure 6(a)). The result of Begg's test was *Pr* > |*z*| = 0.005. Since *Pr* > |*z*| < 0.05, it further indicated that there might be some publication bias in our study results. The test of the combined results of circulating vitamin A concentration and cancer risk showed that Begg's funnel plot was basically symmetrical (Figure 6(b)), and Begg's test result was *Pr* > |*z*| = 0.773. Since *Pr* > |*z*| > 0.05, there was no publication bias. Finally, we performed a sensitivity analysis of the findings (Figures 6(c) and 6(d)), and by removing each study, the pooled OR fluctuated within a certain range without particularly obvious changes, indicating that our findings were more stable.

## 4. Discussion

ROS are metabolic byproducts produced by tissues and cells as they consume ingested oxygen [42]. According to earlier research, ROS is directly linked to the initiation and growth of malignant tumors, and tumor cells create more ROS than normal cells do. Increased ROS levels can lead to DNA, protein, and lipid damage [43, 44]. As a signaling molecule in cancer, ROS has the effects of antiapoptosis, promoting cancer cell metastasis and angiogenesis, and even blocking differentiation [45]. ROS accumulation can damage DNA and lead to genomic instability in cancer, increasing the resistance to chemotherapeutic drugs and the chance of cancer recurrence [46]. The main function of antioxidants is to reduce ROS levels, prevent excessive ROS accumulation, and maintain a dynamic balance between oxidation and reduction [45]. Antioxidants are divided into two main classes, one endogenous and the other exogenous. Endogenous antioxidants are represented by glutathione, which prevents DNA damage and plays an important role in preventing tumor initiation [47, 48]. Exogenous antioxidants are mainly antioxidant substances that people supplement through diet or drugs, of which the vitamin family plays an important role, such as vitamins A, C, and E. We used the meta-analysis to investigate the effect of vitamin A on the risk of the three major cancers in women and to clarify its potential anticancer value.

Vitamin A is a fat-soluble micronutrient and a powerful antioxidant that controls oxidative stress and retards cancer progression [27]. It is crucial for protecting vision, maintaining the integrity of the skin and mucous membranes, maintaining and promoting immunity, promoting growth and development, and maintaining reproductive function [49, 50]. Despite being abundantly found in meat products and a wide range of vegetables and fruits, vitamin A is still insufficient in a significant portion of the global population [51, 52]. Therefore, it is critical to investigate the relationship between vitamin A consumption and the risk of breast, cervical, and ovarian cancer in women, which has significant public health implications. We pooled clinical studies of dietary vitamin A or supplements and circulating vitamin A and the risk of these three cancers by including a total of 49 studies through an extensive search. Further subgroup analysis was done in accordance with the type of cancer, type of study, geographical location, and type of diet or circulation.

Our findings show that high intake of dietary vitamin A and supplements can considerably lower the incidence of three cancers in women. We then subgroup analyzed according to cancer type, study type, geographical location, and diet or supplement. High vitamin A consumption reduces the incidence of ovarian and breast cancer considerably, and the same conclusion can be drawn for both diet and diet plus supplements. Although the cohort study results showed no significant correlation between the two, it may be due to loss to follow-up bias and confounding bias. High vitamin A intake in Asia and North America can greatly lower the risk of these three cancers. Furthermore, in terms of circulating vitamin A, the findings showed that high amounts of circulating vitamin A were not significantly connected with the incidence of the three cancers in women. All results of the subgroup analysis were consistent, and no association was found between them. In summary, higher dietary vitamin A and supplements can considerably lower the incidence of these three cancers and deserve deeper exploration. However, there is no correlation between plasma or serum vitamin A concentrations and the risk of the disease, which is worthy of further verification of this conclusion.

We will further discuss the underlying mechanisms and associated evidence for vitamin A (retinol) in the prevention of cervical, breast, and ovarian cancer. In the past century, retinol and its derivatives have attracted much attention due to their wide range of physiological functions, which can affect cell differentiation, proliferation, and apoptosis, further affect individual growth and aging, and play a vital physiological role in a variety of biological activities [53]. The occurrence and development of tumors are closely related to the dysregulation of retinoid signaling pathway [54]. It has been shown that the bioavailability of intracellular retinoids is regulated by specific cytoplasmic retinol and retinoic acid-binding proteins (CRBPs and CRABPs) [55, 56]. In breast, ovarian, and nasopharyngeal carcinomas, CRBP-1 downregulation is linked to malignant phenotypes [55]. In vitro, CRBP-1 reexpression improved retinol sensitivity while decreasing the viability of ovarian cancer cells [57]. Vitamin A derivatives are crucial cell-permeable signaling agents that activate nuclear receptors to control gene expression [58]. It has also been shown that there is a synergistic effect between carotenoids and vitamin A and chemotherapy agents to promote breast cancer cell apoptosis and inhibit breast cancer cell proliferation [59, 60]. Therefore, retinol derivatives have great potential for the treatment and prevention of malignant tumors and deserve further study of their role in clinical practice, especially as a combination therapy with chemotherapeutic drugs.

Several meta-analyses have previously been conducted to investigate the link between dietary or circulating vitamin A and the incidence of breast, ovarian, or cervical cancer. In 2011, Fulan et al. did a meta-analysis of diet vitamin A intake and breast cancer incidence, discovering that high vitamin A intake lowers the incidence of breast cancer by 17%, which is similar to our findings [61]. The results of Hu et al.'s meta-analysis on the relationship between plasma vitamin A and the incidence of breast malignant tumor and our findings are in agreement. They discovered no association between plasma vitamin A and the incidence of breast malignant tumor [40]. Zhang et al. conducted a meta-analysis of vitamin A and cervical cancer incidence in 2012 and found that high diet consumption of vitamin A was able to lower the incidence of cervical cancer, and the conclusion was consistent with ours. However, they discovered a substantial negative relationship between the incidence of cervical cancer and circulating vitamin A concentrations, while we did not find an association between the two, possibly due to the bias caused by the small number of articles they included [34]. Wang published a meta-analysis of diet vitamin A and ovarian cancer incidence in 2020, which indicated a substantial inverse association between dietary vitamin A and ovarian cancer incidence, which agrees with our study [36]. In conclusion, our study's findings are almost consistent compared with the previous ones, but more literature in recent years is included, which is more persuasive.

We discovered heterogeneity in the combined data of dietary and vitamin A supplements with the risk of the three cancers in women (*I*^2^ = 56.1%, *P* < 0.001), while there was no apparent heterogeneity in the combined data of circulating vitamin A (*I*^2^ = 29.5%, *P* = 0.122). In meta-analysis, heterogeneity is inescapable, and it is our crucial responsibility to identify and investigate its causes. Firstly, when the heterogeneity is large, we choose the random-effects model for effect size combination; secondly, we determine the source of heterogeneity through detailed subgroup analysis. For diet and supplements, we subgroup analyzed by tumor type and found that the heterogeneity of ovarian cancer combined was low, and the heterogeneity of cervical cancer and breast cancer was high; we subgroup analyzed by study type and found that the heterogeneity of the cohort study combined was low, while the heterogeneity of case-control study was high; we subgroup analyzed by diet or supplements and found that the heterogeneity of diet combined was high, while the heterogeneity of supplement was low; finally, we conducted a subgroup analysis by geographic location and discovered that the heterogeneity was high in Asia and low in North America, Oceania, and Europe. Furthermore, various objective factors, such as ethnicity, nutritional structure, economic development status, and HPV vaccination status, may enhance heterogeneity. This may be followed by heterogeneity due to nonuniform methods and details of the study itself and inconsistency between diet and vitamin A concentration assessment tools or scales. The inconsistency in the specific dose limit for high intake and low intake is an important cause of heterogeneity. Therefore, strict and unified inclusion and exclusion criteria, large multicenter randomized controlled trials, or cohort studies are needed to more clarify the relationship between vitamin A and the three cancers in women. We tested the studies used for our meta-analysis for bias by Begg's tests and Begg's funnel plots and found that there may be some publication bias. The reason may be that we only searched the English language database and not the databases of other language kinds, resulting in some studies not being included. It is also possible that there were some unpublished results that we could not retrieve and include. As a result, our findings should be interpreted with care.

Our meta-analysis has several strengths. First, we examined for the first time the relationship between vitamin A intake and the incidence of three forms of malignant tumors in women, taking into account not only dietary but also supplements, as well as serum and plasma; second, we examined the references of relevant meta-analyses and reviews on the basis of searching databases to prevent missing studies as much as possible; third, our meta-analysis includes 49 papers with a large number of controls and cases to acquire more credible results; fourth, we conducted an in-depth subgroup analysis based on type of cancer, type of study, diet or supplements, serum or plasma, and geographical location; and fifth, all of the selected researches were adjusted for multivariate covariates to reduce confounding bias.

We acknowledge that this meta-analysis has the following insufficiencies. Firstly, the language of the included research was confined to English, which may cause bias; secondly, the studies had some publication bias, although we explored the source of bias; thirdly, although the included studies were adjusted for covariates, there may still be the effect of other confounders; fourthly, although the specific dose or concentration ingested in each study was stated, a meta-analysis of dose response was not performed; and fifthly, the food and vitamin A conversion scales or tools were not uniform, and the specific dose of high and low intake was different and not uniform.

A high vitamin A consumption can lower the incidence of cervical, breast, and ovarian cancer. Although we recommend that women prevent these three cancers by appropriately increasing vitamin A, we must explain the toxic side effects of huge doses. Acute poisoning occurs when adults take more than 1 million IU of vitamin A each time and children take more than 300,000 IU each time. High levels of vitamin A (100,000-200,000 IU) is frequently used in relevant clinical trials, which raises our concerns about the toxicity of excessive intake of vitamin A. Cases have been reported in which excessive intake of vitamin A can lead to acute toxic reactions, including headache, nausea, vomiting, diarrhea, blurred vision, depression and anxiety, and other symptoms of nerve damage [62–65]. In addition, studies have shown teratogenic effects in pregnant women ingesting large amounts of vitamin A [66]. Therefore, within the range of acceptable doses, we advise adequately increasing vitamin A consumption, particularly by increasing dietary intake of fruits, vegetables, and meat food.

## 5. Conclusion

In North American and Asian populations, higher dietary consumption of vitamin A or supplements lowers the incidence of three cancers in women, with breast and ovarian cancers being more significant. However, high circulating vitamin A concentrations were not significantly linked to the incidence of the three malignancies. Moderate increases in dietary vitamin A intake or supplements are recommended to prevent the development of these cancers in high-risk groups for these three cancers. From this point of view, vitamin A, as one of the typical antioxidants, has a promising medical application, and multicenter prospective cohort studies or large randomized controlled trials are encouraged to verify the above conclusions.

## Figures and Tables

**Table 1:** Characteristics of included studies.

Author Year Country	Age	Type of cancer	Type of study	Sample size	Diet/serum/plasma/supplements	Dose: highest comparison lowest	OR (95%Cl)	Adjustment for covariates.	NOS score
Fernandez-Lazaro et al. [67] 2021 Spain	Mean age: 35.1	Breast cancer	Cohort study	9983/107	Diet	>2282 vs. <1387 (mcg/d)	0.94 (0.59–1.50)	Age, smoking, alcohol intake, BMI, height, reproductive history, and family history of breast cancer.	7
Tamimi et al. [68] 2005 USA	Mean age: 57	Breast cancer	Case-control study	969/969	Plasma	five-fold vs. one-fold	0.78 (0.56–1.07)	Age, menstrual history, body mass index, family history of breast disease, reproductive history, alcohol consumption, and smoking.	7
Maillard et al. [69] 2010 France	Mean age: 56.8	Breast cancer	Case-control study	366/720	Serum	five-fold vs. one-fold	0.85 (0.53–1.35)	Alcohol, height, use of menopausal hormones, educational level, age and parity at birth, family history of breast cancer, and history of benign breast disease.	8
Epplein et al. [70] 2009 USA	Mean age: 66	Breast cancer	Case-control study	286/535	Plasma	≥1,188.0 vs ≤842.6 (ng/ml)	1.13 (0.73–1.76)	Smoking, ethnicity, body mass index, alcohol use, age at first birth, number of full-term pregnancies, age at menarche, and age at menopause.	8
Bakker et al. [71] 2016 Europe	Mean age: 50	Breast cancer	Case-control study	1502/1502	Plasma	five-fold vs. one-fold	ER (-): 1.65 (0.97–2.81) ER (+): 1.02 (0.64–1.63)	BMI, height, age at menarche, reproductive history, smoking, alcohol use, education, energy intake, and blood sampling time.	8
Longnecker et al. [72] 1997 USA	65-74	Breast cancer	Case-control study	3285/8736	Diet	≥12000 vs. <3000 (IU/day)	0.40 (0.15–1.05)	Age, reproductive history, body mass index, age at menarche, education, family history of breast cancer, smoking, and alcohol consumption.	6
Sato et al. [73] 2002 USA	51.3	Breast cancer	Case-control study	244/244	Serum	≥73.0 vs. <46.2 (*μ*g/dl)	0.97 (0.53–1.80)	Family history of breast cancer, age at birth, age at menarche, alcohol use, smoking status, body mass index, lactation, and education.	6
Cho et al. [74] 2003 USA	25-42	Breast cancer	Cohort study	90655/714	Diet	Median intake 21,916 (IU/day) vs. 5639 (IU/day)	0.92 (0.72–1.17)	Smoking status, alcohol consumption, body mass index, height, age at menarche, oral contraceptive use, family history of breast cancer, reproductive history, menopausal status, and energy intake.	6
Zhang et al. [75] 2009 China	Mean age: 47	Breast cancer	Case-control study	438/438	Diet	four-fold vs. one-fold	0.31 (0.20–0.48)	Age at menarche, BMI, family history of breast cancer in first degree relatives, history of benign breast disease, physical activity, and smoking.	7
Do et al. [76] 2003 Korean	20-69	Breast cancer	Case-control study	224/250	Diet+supplements	≥2902.72 vs. <1773.41 (RE)	0.63 (0.39–0.98)	Age at menarche, total menstrual cycle, pregnancy, total number of full-term deliveries, total lactation period, family history of breast cancer, and BMI.	7
London et al. [77] 1992 USA	Mean age: 64	Breast cancer	Case-control study	377/403	Diet/serum	Diet: median 10916 vs. 639 (IU/day) Serum: median 2.70 vs. 1.47 (*μ*mol/l)	Diet: 0.7 (0.4–1.3) Serum: 1.0 (0.6–1.7)	Age, alcohol intake, age at birth, parity, family history of breast cancer, age at menopause, age at menarche, and weight.	6
Hulten et al. [78] 2001 Sweden	49-65	Breast cancer	Case-control study	201/290	Plasma	four-fold vs. one-fold	0.9 (0.5–1.4)	Age, alcohol intake, parity, family history of breast cancer, age at menopause, age at birth, physical activity, and weight.	6
Zhang et al. [37] 1999 USA	33-60	Breast cancer	Cohort study	83234/2697	Diet	Median 17073 vs. 5293 (IU/day)	Premenopausal: 0.82 (0.65–1.04) Postmenopausal: 1.03 (0.89–1.19)	Age, parity, family history of breast cancer, weight, age at menopause, and use of postmenopausal hormones.	8
Rohan et al. [79] 1993 Canada	40-59	Breast cancer	Case-control study	519/1182	Diet/supplements	Diet: five-fold vs. one-fold Supplements: three-fold vs. one-fold	Diet: 0.80 (0.55–1.17) Supplements: 0.70 (0.42–1.15)	Age, energy intake, age at menarche, surgical menopause, age at first birth, years of education, family history of breast cancer, and history of benign breast disease.	6
Kushi et al. [80] 1996 USA	Mean age: 61	Breast cancer	Cohort study	34387/879	Diet/supplements	Diet: ≥20343 vs. ≤7254 (IU/day) Supplements: >10,000 vs. 0 (IU/day)	Postmenopausal: Diet: 1.26 (0.88–1.80) Supplements: 0.74 (0.47–1.18)	Age, energy intake, menstrual history, marriage and childbearing history, body mass index, family history of breast cancer, alcohol consumption, and education.	7
Negri et al. [81] 1996 Italy	/	Breast cancer	Case-control study	2569/2588	Diet	≥2361 vs. ≤706 (*μ*g/day)	0.73 (0.6–0.9)	Age, weight, family history of breast disease, reproductive history, smoking, alcohol, and energy intake.	8
Bohlke et al. [82] 1999 USA	Mean age: 56.4/54.4	Breast cancer	Case-control study	820/1548	Diet	≥2120.7 vs. ≤659.1 (*μ*g/day)	0.89 (0.65–1.23)	Age, place of birth, body mass index, reproductive history, menstrual history, and energy intake.	8
Verhoeven et al. [83] 1997 Netherlands	55-69	Breast cancer	Cohort study	62573/650	Diet	≥0.766 vs. ≤0.229 (mg/day)	1.24 (0.83–1.83)	Age, energy intake, alcohol, family history of breast cancer, menstrual history, and reproductive history.	8
Dorjgochoo et al. [84] 2009 China	40-70	Breast cancer	Case-control study	365/726	Plasma	four-fold vs. one-fold	1.20 (0.80–1.81)	Age, education, occupation, menstrual history, reproductive history, physical exercise, smoking, alcohol consumption, family history of breast cancer, and total energy intake.	7
Dorgan et al. [85] 1998 USA	Mean age: 58	Breast cancer	Case-control study	105/203	Serum	≥2.31 vs. ≤1.67 (*μ*mol/l)	0.9 (0.4-2.0)	Serum total cholesterol concentration, smoking, and body mass index (kg/m^2^).	6
Kabat et al. [86] 2009 USA	Mean age: 62	Breast cancer	Cohort study	5450/190	Serum	≥0.65 vs. <0.53 (*μ*g/ml)	1.00 (0.68–1.48)	Age, education, race, body mass index, reproductive history, menstrual history, alcohol, energy intake, and family history of breast cancer.	8
Ching et al. [87] 2002 Australia	Mean age: 54	Breast cancer	Case-control study	153/151	Serum	≥2.50 vs. ≤1.90 (*μ*mol/l)	0.53 (0.28–1.01)	Age at menarche, parity, alcohol intake, and total fat intake.	7
Michels et al. [88] 2001 Swedish	40-76	Breast cancer	Cohort study	508267/1271	Diet	≥1.51 vs. ≤0.52 (mg/day)	1.00 (0.83–1.20)	Age, family history of breast cancer, height, body mass index, education, reproductive history, energy intake, and alcohol, fiber intake.	8
Hunter et al. [89] 1993 England	34-59	Breast cancer	Cohort study	89494/1439	Diet+supplements	≥17640 vs. <6630 (IU/day)	0.84 (0.71–0.98)	Age, follow-up time, ethnicity, family history of breast cancer, body mass index, education, reproductive history, energy intake, and alcohol.	8
Mignone et al. [90] 2009 USA	/	Breast cancer	Case-control study	5707/6389	Diet	five-fold vs. one-fold	0.84 (0.74–0.94)	Age, status, family history of breast cancer, reproductive history, alcohol use, education, menstrual history, body mass index, smoking, and hormone replacement therapy.	7
Pantavos et al. [31] 2015 Netherlands	>55	Breast cancer	Cohort study	53209/199	Diet	/	0.98 (0.69–1.39)	Age, BMI, educational level, family history of breast cancer, smoking and alcohol consumption, reproductive history, and fiber intake.	8
Katsouyanni et al. [91] 1988 Greek	Mean age: 54.7/53.7	Breast cancer	Case-control study	120/120	Diet	≥12482 vs. <6868 (IU/day)	0.40 (0.17–0.93)	Age at first birth, menopausal status, age at menarche, age at menopause, residence, marital status, and other significant (10% level) nutrients.	7
Marubini et al. [92] 1988 Italy	30-65	Breast cancer	Case-control study	214/215	Diet/serum	five-fold vs. one-fold	Plasma: 2.0 (1.0–4.0) Diet: 0.7 (0.4–1.5)	Age, triglycerides, and cholesterol.	7
Rohan et al. [93] 1988 Australia	20-74	Breast cancer	Case-control study	451/451	Diet	>1445.8 vs. ≤245.6 (*μ*g/day)	1.19 (0.78–1.82)	Family history of breast cancer, menstrual history, oral contraceptives or not, smoking, and years of education.	7
González et al. [94] 2011 Europe	35-70	Cervical cancer	Cohort study	299649/1070	Diet	>949.23 vs. <287.24 (*μ*g/d)	1.01 (0.83–1.22)	BMI, energy intake, smoking, alcohol use, physical activity, marriage and childbearing history, and education.	8
Shannon et al. [95] 2002 Thailand	/	Cervical cancer	Case-control study	134/384	Diet	≥6000 vs. ≤1400 (IU/day)	0.32 (0.13–0.83)	Smoking, drinking, HPV infection, body mass index, education, history of sexual intercourse, and reproductive history.	6
Ghosh et al. [38] 2008 USA	Mean age: 52	Cervical cancer	Case-control study	239/979	Diet	≥12768 vs. ≤7420 (IU/day)	0.47 (0.30–0.73)	Adjusted for age, education level, income, smoking status, body mass index, marriage and childbearing history, family history of cervical cancer, and energy intake.	6
Kim et al. [96] 2010 Korea	/	Cervical cancer	Case-control study	144/288	Diet	≥999 vs. ≤609 (RE/day)	0.36 (0.19–0.69)	Adjusted for age, smoking status, alcohol consumption status, exercise, family history, body mass index, and human papillomavirus infection status.	6
Guo et al. [97] 2015 China	Mean age: 47.4/46.3	Cervical cancer	Case-control study	458/742	Diet/serum	Serum: ≥92 vs. ≤25 (*μ*g/day) Diet: ≥450 vs. ≤80 (*μ*g/day)	Serum: 0.85 (0.61–1.19) Diet: 1.06 (0.75–1.50)	Age, body mass index, marital status, education, family history of cancer, HPV infection, passive smoking, current alcohol consumption, calcium supplementation, physical activity, and daily energy intake.	7
Brock et al. [98] 1988 Australia	18-65	Cervical cancer	Case-control study	117/196	Plasma/diet	Plasma: >68 vs. ≤45 (*μ*g/dl) Diet: >2248 vs. ≤565 (*μ*g/day)	Plasma: 0.4 (0.1-1.3) Diet: 1.2 (0.4–3.60)	Number of sexual partners, age at first intercourse, smoking, use of oral contraceptives, and other listed risk factors (i.e., carotene, vitamin C, folic acid, retinol, and total energy).	8
Verreau et al. [99] 1989 USA	Mean age: 44.6/43	Cervical cancer	Case-control study	189/227	Diet	>805 vs. ≤248 (*μ*g/day)	1.1 (0.5–2.2)	Age, education, smoking, oral contraceptive use, history of cervicovaginal infection, number of sexual partners, and total energy intake.	7
Slattery et al. [100] 1990 USA	20-59	Cervical cancer	Case-control study	266/408	Diet	≥13876 vs. <6383 (IU/day)	0.71 (0.43–1.2)	Age, education, smoking, alcohol use, family history, and sexual partner.	7
Potischman et al. [101] 1991 USA	/	Cervical cancer	Case-control study	387/670	Serum	>56.1 vs. ≤35.9 (*μ*g/dl)	1.28 (0.8–2.1)	Age, age at first sexual intercourse, number of sexual partners, reproductive history, whether HPV infection, family history, triglyceride, and cholesterol.	7
Herrero et al. [102] 1991 USA	Mean age: 46.5	Cervical cancer	Case-control study	748/1411	Diet	>1830 vs. ≤217 (*μ*g/day)	1.13 (0.8–1.5)	Age, age at first sexual intercourse, number of sexual partners, reproductive history, whether HPV infection, family history, triglyceride, and cholesterol.	7
Cramer et al. [103] 2001 USA	/	Ovarian cancer	Case-control study	549/516	Diet	≥18721 vs. <6478 (IU/day)	0.77 (0.51–1.18)	Age, study site, marriage and childbearing history, family history, BMI, and energy intake.	8
Silvera et al. [41] 2006 USA	Mean age: 48.6	Ovarian cancer	Case-control study	487/264	Diet/diet+supplements	>11,534 vs. <6,589 (IU/d) >11,560 vs. <6,595 (IU/d)	Diet: 0.77 (0.52–1.14) Diet+supplements: 0.79 (0.53–1.16)	Age, smoking, alcohol use, menopausal status, BMI, education, and physical activity.	7
Bertone et al. [104] 2001 USA	50-79	Ovarian cancer	Case-control study	327/3129	Diet	≥18000 vs. <4600 (IU/d)	0.84 (0.57–1.2)	Age, BMI, menstrual history, marriage and childbearing history, family medical history, physical activity, race, religion, smoking, and alcohol.	6
McCann et al. [105] 2001 USA	20-87	Ovarian cancer	Case-control study	496/1425	Diet	>14204 vs. ≤5917 (IU/day)	0.66 (0.45–0.98)	Age, education, region of residence, regularity of menstruation, family history of ovarian cancer, parity, age at menarche, oral contraceptive use, and total energy intake.	6
Tung et al. [106] 2005 USA	Mean age: 54.8	Ovarian cancer	Case-control study	558/607	Diet	four-fold vs. one-fold	0.61 (0.42–0.89)	Age, ethnicity, site, education, oral contraceptive use, parity, and tubal ligation.	7
Tzonou et al. [107] 1993 Greece	/	Ovarian cancer	Case-control study	189/200	Diet	≥11000 vs. <7000 (IU/day)	0.87 (0.73–1.03)	Age, years of education, parity, age at first birth, menopausal status, and energy intake.	8
Salazar-Martinez et al. [108] 2002 Mexico	20-79	Ovarian cancer	Case-control study	84/629	Diet	≥718 vs. ≤521 (RE)	0.52 (0.28–0.95)	Age, total energy intake, number of live births, recent changes in weight, physical, activity (METs), and diabetes.	6
Chang et al. [109] 2007 USA	/	Ovarian cancer	Cohort study	97275/280	Diet	>695 vs. ≤233 (*μ*g/day)	1.17 (0.52–2.66)	Ethnicity, total energy intake, parity, oral contraceptive use, strenuous exercise, wine consumption, and menopausal status/hormonal therapy.	7
Jeong et al. [110] 2009 Korea	Mean age: 52.8/52.1	Ovarian cancer	Case-control study	45/135	Plasma	>134 vs. ≤23.8 (*μ*g/dl)	0.45 (0.21–0.98)	Education level, BMI, menopause, number of births, oral contraceptive use, smoking status (former vs. never), and alcohol use.	7
Bidoli et al. [111] 2001 Italy	Mean age: 56/57	Ovarian cancer	Case-control study	1031/2411	Diet	five-fold vs. one-fold	1.1 (0.8–1.4)	Age, site, year of interview, education, body mass index, parity, oral contraceptive use, occupational physical activity, and energy intake.	8

**Table 2:** Metaresults of dietary or supplements and circulating vitamin A and risk of breast, cervical, and ovarian cancer in women.

	Studies (*n*)	OR	95% CI	*P* value	Model	Heterogeneity	
						*I* ^2^	*P* value
Sum of three cancers (dietary or supplements)	37	0.83	0.76-0.90	0.001	Random	56.1%	0.001
Breast cancer (dietary or supplements)	19	0.84	0.76-0.93	0.001	Random	59.9%	0.001
Cervical cancer (dietary or supplements)	9	0.77	0.59-1.12	0.071	Random	69.6%	0.001
Ovarian cancer (dietary or supplements)	9	0.81	0.72-0.92	0.001	Random	20.7%	0.253
Sum of three cancers (circulating)	16	0.96	0.86-1.09	0.552	Fix	29.5%	0.122
Breast cancer (circulating)	12	1.00	0.87-1.14	0.944	Fix	20.2%	0.239
Cervical cancer (circulating)	3	0.93	0.71-1.22	0.616	Fix	44.8%	0.163
Ovarian cancer (circulating)	1	0.45	0.21-0.97	0.042	—	—	—

## Data Availability

Data resulting from this study may be obtained from the corresponding author upon reasonable request.
